# Discovery of Novel Inhibitors From Medicinal Plants for V-Domain Ig Suppressor of T-Cell Activation

**DOI:** 10.3389/fmolb.2021.716735

**Published:** 2021-10-26

**Authors:** Iqra Muneer, Sajjad Ahmad, Anam Naz, Sumra Wajid Abbasi, Adel Alblihy, Abdulaziz A. Aloliqi, Faris F. Aba Alkhayl, Faris Alrumaihi, Sarfraz Ahmad, Youness El Bakri, Muhammad Tahir Ul Qamar

**Affiliations:** ^1^ School of Life Sciences, University of Science and Technology of China, Hefei, China; ^2^ Department of Health and Biological Sciences, Abasyn University, Peshawar, Pakistan; ^3^ Institute of Molecular Biology and Biotechnology, The University of Lahore, Lahore, Pakistan; ^4^ NUMS Department of Biological Sciences, National University of Medical Sciences, Rawalpindi, Pakistan; ^5^ Medical Center, King Fahad Security College (KFSC), Riyadh, Saudi Arabia; ^6^ Department of Medical Biotechnology, College of Applied Medical Sciences, Qassim University, Buraydah, Saudi Arabia; ^7^ Department of Medical Laboratories, College of Applied Medical Sciences, Qassim University, Buraydah, Saudi Arabia; ^8^ Department of Chemistry, Faculty of Science, University of Malaya, Kuala Lumpur, Malaysia; ^9^ Department of Theoretical and Applied Chemistry, South Ural State University, Chelyabinsk, Russia; ^10^ College of Life Science and Technology, Guangxi University, Nanning, China

**Keywords:** VISTA, breast cancer, medicinal plant, phytochemical, MD simulation

## Abstract

V-domain Ig suppressor of T cell activation (VISTA) is an immune checkpoint and is a type I transmembrane protein. VISTA is linked to immunotherapy resistance, and it is a potential immune therapeutic target, especially for triple-negative breast cancer. It expresses at a high concentration in regulatory T cells and myeloid-derived suppressor cells, and its functional blockade is found to delay tumor growth. A useful medicinal plant database for drug designing (MPD3), which is a collection of phytochemicals from diverse plant families, was employed in virtual screening against VISTA to prioritize natural inhibitors against VISTA. Three compounds, Paratocarpin K (PubChem ID: 14187087), 3-(1H-Indol-3-yl)-2-(trimethylazaniumyl)propanoate (PubChem ID: 3861164), and 2-[(5-Benzyl-4-ethyl-1,2,4-triazol-3-yl)sulfanylmethyl]-5-methyl-1,3,4-oxadiazole (PubChem ID: 6494266), having binding energies stronger than −6 kcal/mol were found to have two common hydrogen bond interactions with VISTA active site residues: Arg54 and Arg127. The dynamics of the compound–VISTA complexes were further explored to infer binding stability of the systems. Results revealed that the compound 14187087 and 6494266 systems are highly stable with an average RMSD of 1.31 Å. Further affirmation on the results was achieved by running MM-GBSA on the MD simulation trajectories, which re-ranked 14187087 as the top-binder with a net binding energy value of −33.33 kcal/mol. In conclusion, the present study successfully predicted natural compounds that have the potential to block the function of VISTA and therefore can be utilized further in experimental studies to validate their real anti-VISTA activity.

## Introduction

Immunotherapy has turned into an important pillar of cancer treatment due to the successful blocking of the programmed cell death protein 1 (PD-1) and its ligand-programmed death-ligand 1 (PD-L1) immune checkpoints. Immune checkpoint receptors control the duration and intensity of immune response by inhibiting T cell activation (Tang et al., 2018). Several immune checkpoint proteins have been discovered, such as PD-1/PD-L1, TIGIT, VISTA, cytotoxic T lymphocyte antigen-4 (CTLA-4), TIM3, BTLA, and LAG3. PD-1 inhibitors such as nivolumab, pembrolizumab, and cemiplimab and the human IgG1 k anti-CTLA-4 monoclonal antibody ipilimumab have been approved by the Food and Drug Administration (FDA). These approved drugs have become successful cancer therapies. However, the relatively low response rate of current immunotherapy drugs (less than 30%) is still a serious challenge, and efforts are needed to identify and overcome other immunosuppressive pathways (Ventola, 2017).

In the ever-expanding list of immune checkpoints, VISTA (V-domain immunoglobulin suppressor of T cell activation) is considered to be an important regulator of the immune system. VISTA immune checkpoint protein is a type 1 transmembrane protein that is encoded by the C10orf54 gene (Wang et al., 2011). VISTA is part of the B7 family consisting of a single extracellular N-terminal Ig-V domain, a stalk with approximately 30 amino acids, a transmembrane domain, and a cytoplasmic domain (Flies et al., 2011). The closest homolog of VISTA in the B7 family is PD-L1, sharing 23% sequence identity. VISTA is highly expressed in tumor-infiltrating lymphocytes. VISTA is also expressed in CD4^+^ and CD8^+^ cells, where it negatively regulates T cell responses (Borggrewe et al., 2018; ElTanbouly et al., 2019). It has also been observed that VISTA is highly expressed in breast cancer as compared to other cancer types, indicating that targeting VISTA may benefit breast cancer immunotherapy (Xie et al., 2020). Interestingly, expression of VISTA has also been observed in different cancer types such as breast invasive carcinoma (BRCA), invasive ductal carcinoma (IDC), bladder urothelial carcinoma (BLCA), colon adenocarcinoma (COAD), kidney chromophobe (KICH), lung squamous cell carcinoma (LUSC), uterine carcinosarcoma (UCS), and skin cutaneous melanoma (SKCM). Recently, it has been reported that VISTA is the acidic pH selective ligand of PSGL-1, which means that it may engender resistance to antitumor immune response (Johnston et al., 2019). Research on a variety of clinical samples, autoimmune disease models, and tumor models has shown that VISTA has a key regulatory effect on the immune system and has the potential to be used as a therapeutic or combined drug target. These findings indicated that the high expression of VISTA on tumor cells in about 20% of NSCLC specimens can prove the feasibility of targeting VISTA for cancer therapy (Cuzick et al., 2015). Clinical studies have shown that the expression of VISTA is upregulated in oral squamous cell carcinoma and gastric cancer (Böger et al., 2017; Wu et al., 2017). After ipilimumab therapy, the VISTA immune checkpoint has also increased in patients with prostate cancer (Gao et al., 2017; Kakavand et al., 2017). In addition, previous studies have shown that VISTA is highly expressed in the immune cell subsets of human pancreatic cancer patients (Xie et al., 2018b).

Currently, compound CA-170 is undergoing phase I clinical trial for advanced tumors and lymphoma. CA-170 exhibits powerful activity to stop the lymphocyte proliferation and effector functions inhibited by VISTA proteins. CA-170 also exhibits selectivity for other immune checkpoints such as CTLA4, BTLA, and LAG3. These nonclinical data provide a strong basis for the clinical development of CA-170 (Sasikumar and Ramachandra, 2018; Wang et al., 2018; Blevins et al., 2019). In this study, we performed molecular docking to select natural drugable molecules from medicinal plants which may act as antagonists against VISTA. Molecular dynamics (MD) studies were carried out to further verify the binding of natural leads with VISTA protein.

## Materials and Methods

### Phytochemical’s Library Retrieval and Filtration

The MPD3 database’s (https://www.bioinformation.info/) (Mumtaz et al., 2017) diverse and ready-to-dock library of phytochemicals was retrieved and filtered for lead-like molecules. Lead molecules may serve as the starting point for further structural optimization and have the best chance to become good drug candidates. The lead molecule filtration was accomplished through the online FAF-Drugs4 server (Lagorce et al., 2017). The different physicochemical parameters applied during filtration include the following: molecular weight (150–400 kDa), logP (−3 to 4), hydrogen bond donor number (≤4), hydrogen bond acceptor number (≤7), TPSA (≤160), rotatable bonds (≤9), rigid bonds (≤30), rings (≤4), maximum ring size of system (≤18), number of heteroatoms (1–15), carbon number (3–35), charges (≤4), ratio of H/C (0.1–1.1), total charge (−4 to 4), and stero centers (≤2). These parameters were applied in accordance with Lipinski’s (Lipinski et al., 1997), Veber’s (Veber et al., 2002), and Egan’s (Egan et al., 2000) rules, to screen out the most promising hits for downward analysis.

### Docking Studies

The human VISTA extracellular domain crystal structure present in the RCSB PDB database with the PDB ID: 6OIL was retrieved and processed in UCSF Chimera (Pettersen et al., 2004) for the molecular docking process. The structure was prepared first by removing co-crystalized water molecules and the NAG ligand, and then missing hydrogen atoms were added and minimized for energy *via* two algorithms, conjugate gradient and steepest descent, keeping the step size of 0.02 Å. Autodock Vina (Trott and Olson, 2010) was used to dock the ligand library against VISTA. We set the number of binding modes to 20 and exhaustiveness to 20. The grid dimensions were 40 × 40 × 40 (x, y, z), focused on the binding site of the VISTA native ligand along the XYZ dimension of 28.474 × 31.645 × 34.012 Å. Each docked pose was ranked using the Vina empirical scoring function where the most negative binding energy implies the most stable complex. The top 20 docked ligands with the lowest docking energy were considered for further analyses. Protein–ligand interactions were visualized using Pymol (DeLano, 2002).

### Analysis of Complex Dynamics Using MD Simulations

The FF14SB force field of the AMBER 18 (Case et al., 2012) molecular dynamics (MD) simulation package was used for preparation of protein parameters, while its GAFF force field was used for generating ligand parameters (Wang et al., 2004). The whole system was solvated in the water box (TIP3P), considering the padding distance of 12 Å (Jorgensen et al., 1983). Particle mesh Ewald (PME) was employed for processing long-range electrostatic interactions (Darden et al., 1993), and for the nonbonded interactions, the distance cutoff was allowed to be 10 Å. The SHAKE algorithm was used to constrain the bonds involving hydrogen (Ryckaert et al., 1977). All the systems were subjected to energy minimization by running 1,000 steps of the steepest descent and conjugate gradient algorithms. Temperature of each system was equilibrated to 300 K using NVT for a time period of 20 picoseconds (ps), gradually. Afterward, the system equilibration was achieved using NPT ensemble. Finally, a production run of 50 ns was performed, and each trajectory was saved after every 2 fs. Root mean square deviation (RMSD) and root mean square fluctuation (RMSF) analysis of all trajectories was performed to check the system stability by using module CPPTRAJ (Roe and Cheatham, 2013).

### Free Energies Estimation by AMBER MMPBSA.py

The MM-GBSA method in AMBER 18 was used to estimate free energies binding for complexes (Miller et al., 2012). 100 snapshots separated at equal intervals were collected from MD trajectories to carry out the binding free energy calculations. In MM-GBSA, estimation of the net binding free energy ( $\Delta G_{bind}$ ) is done as follows:

$$\Delta G_{binding}=\Delta G_{complex\;\left(receptor+ligand\right)}-\left(\Delta G_{receptor}+\Delta G_{ligand}\right).$$ (1)

In Equation 1, $\Delta G_{complex}$ is complex free energy, $\Delta G_{receptor}$ is receptor free energy, and $\Delta G_{ligand}$ is ligand free energy. The free energy of the above terms can be gained by using the equations given below:

$$\Delta G=\Delta G_{gas}+\Delta G_{sol}-T\Delta S_{s}$$ (2)

$$\Delta G_{gas}=\Delta E_{elec}+\Delta E_{vdw}.$$ (3)

$$\Delta G_{sol}=\Delta G_{GB}+\Delta G_{SA}.$$ (4)

In Equation 2, ΔG is the free energy. TΔS corresponds to entropy energy. In Eq. 3, the electrostatic interaction energy $\left(\Delta E_{elec}\right)$ and van der Waals interaction energy $\left(\Delta E_{vdw}\right)$ collectively correspond to the molecular mechanics energy in the gas phase $\left(\Delta G_{gas}\right)$ . The polar contribution $\left(\Delta G_{GB}\right)$ and the nonpolar contribution $\left(\Delta G_{SA}\right)$ result in solvation free energy $\left(\Delta G_{sol}\right)$ . The MM-PBSA method takes more time than MM-GBSA. Hou T et al. described that to calculate the relative $\Delta G_{bind}$ , MM-GBSA is better in terms of result accuracy than MM-PBSA (Gohlke et al., 2004; Hou et al., 2011; JyrkkäRinne et al., 2012). This approach has been extensively employed in protein–protein interaction and protein–ligand binding studies (Alamri et al., 2021; Tahir ul Qamar et al., 2021).

### Computational Prediction of Compound Pharmacokinetics

The selected compounds were also subjected to different predictions such as drug-likeness, lead-likeness, pharmacokinetics, medicinal chemistry, and toxicity to guide synthetic chemists in optimizing the structure to be successful in clinical studies. Computational predictions of the compound parameters as discussed above were done using the SWISSADME server (Daina et al., 2017).

## Results and Discussion

### Retrieval of Lead Compounds From MPD3 Database

**FIGURE 1:**
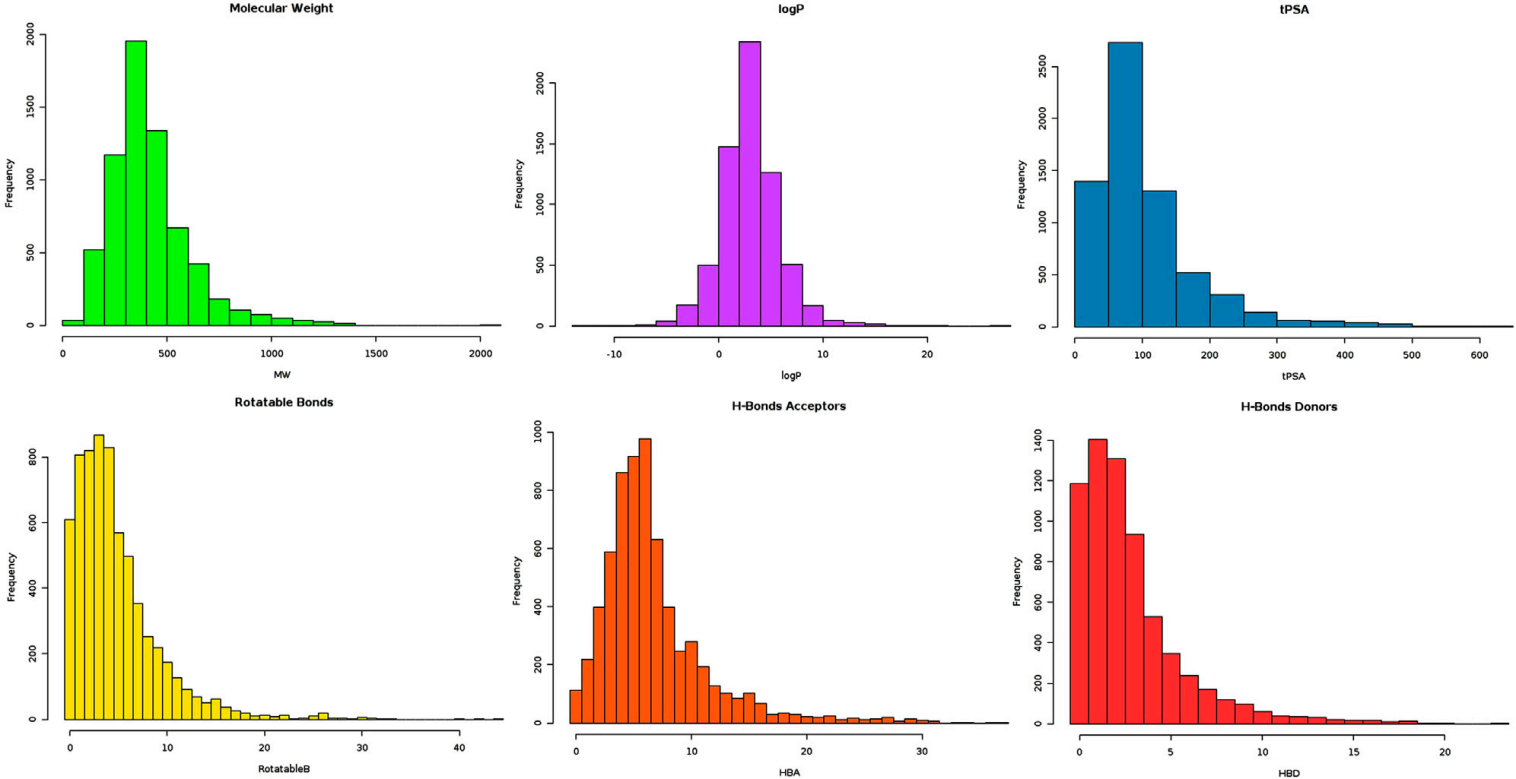
Graphical illustration of FAF-Drugs4 server output, highlighting distribution of lead-like compounds on different parameters (bars of different colors are associated with different parameter results).

The proposed research involves virtual screening of the MPD3 database against VISTA protein, followed by MD simulations and MM-GBSA methods. MPD3 is a collection of uniquely retrieved phytochemical compounds with reported therapeutic potential. The natural compounds were preferred because they are safer, possess better pharmacokinetics, and are easy to test in further experimental studies (Riaz et al., 2017). The lead-like compounds from MPD3 were considered to be therapeutically useful in the drug discovery process, as such compounds have improved selectivity, potency, and medicinal chemistry parameters (Hughes et al., 2011). Additionally, such compounds’ structures can be easily optimized to get the desired biological activity. Previously, only Li et al. (2020) and Gabr and Gambhir (2020) reported small-molecule inhibitors against VISTA. Thus, lead-like natural compounds from MPD3 were retrieved (Figure 1). In total, 1,634 molecules were able to fulfill the criteria of lead-like compounds. Theses 1,634 compounds were used for subsequent docking studies.

### Molecular Docking of CA-170 Into VISTA Immune Checkpoint

**FIGURE 2:**
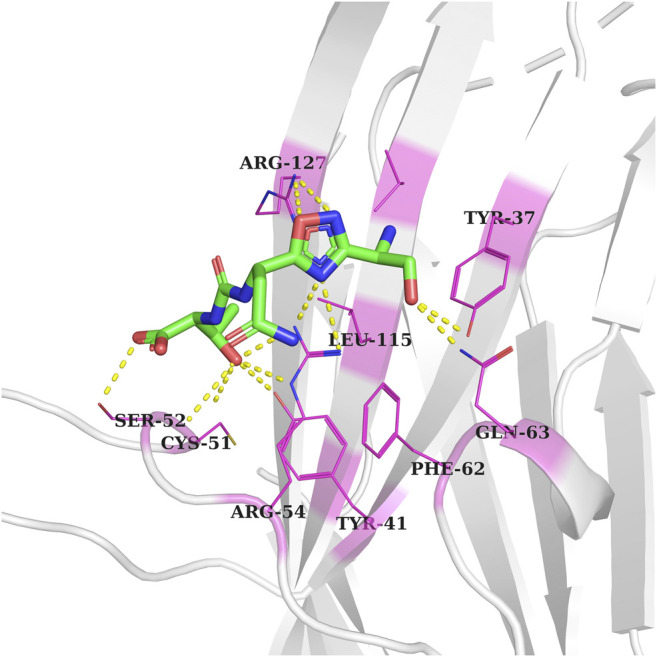
Docked conformation of the CA-170 inhibitor in the VISTA active pocket (gray color). Interacting residue of VISTA is shown as pink-colored lines-cartoons and labeled. Meanwhile, hydrogen bonding between residues is represented by yellow-colored dotted lines.

The CA-170 small molecule has been reported as a dual inhibitor of PDL1/L2 and VISTA in order to treat advanced solid tumors and lymphomas. CA-170 is under phase II clinical trials for head and neck/oral cavity cancer, MSI-H positive cancers, lung cancer, and Hodgkin lymphoma in India. Its exact chemical structure has not been disclosed; however, some studies suggested that CA-170 is a peptidomimetic compound, composed of D-asparagine, L-serine, and L-threonine (Sasikumar et al., 2018; Musielak et al., 2019). Recently, the X-ray structure of the human VISTA extracellular domain has been solved at a resolution of 1.85 Å (PDB ID: 6OIL). VISTA is implicated in different cancers, including breast (Zong et al., 2020), skin (melanoma) (Kakavand et al., 2017), prostate (Gao et al., 2017), colon (Xie et al., 2018a), pancreatic (Xie et al., 2018b), ovarian (Mulati et al., 2019), and lung cancer (Villarroel-Espindola et al., 2018). A single-point mutation study to find the essential residues involved in the interaction of anti-VISTA antibody VSTB showed that three residues, Arg54, Phe62, and Gln63, are essential for the binding of VISTA to VSTB. The latter suggested that targeting these residues would be a valuable approach to inhibiting the VISTA immune checkpoint. In order to predict the binding pocket of CA-170 within the VISTA immune checkpoint, a flexible structure-based docking of CA-170 (PubChem ID: 123843830) using Autodock vina software was performed, following the same protocol as mentioned in the methodology. The grid box which represents the docking search area was centered to cover three key residues (Arg54, Phe62, and Gln63). Interestingly, the top pose of CA-170 with the lowest binding energy was forming hydrogen bonds with the Tyr41, Tyr37, Cys51, Ser52, and Arg127, including two crucial residues, Arg54 and Gln63 (Figure 2). Previous study indicated that a single-point mutation of Arg54 into Ala led to the abolition of the binding of anti-VISTA antibody VSTB to VISTA (Mehta et al., 2019). These results validated the docking protocol being applied in this study.

### Virtual Screening

**FIGURE 3:**
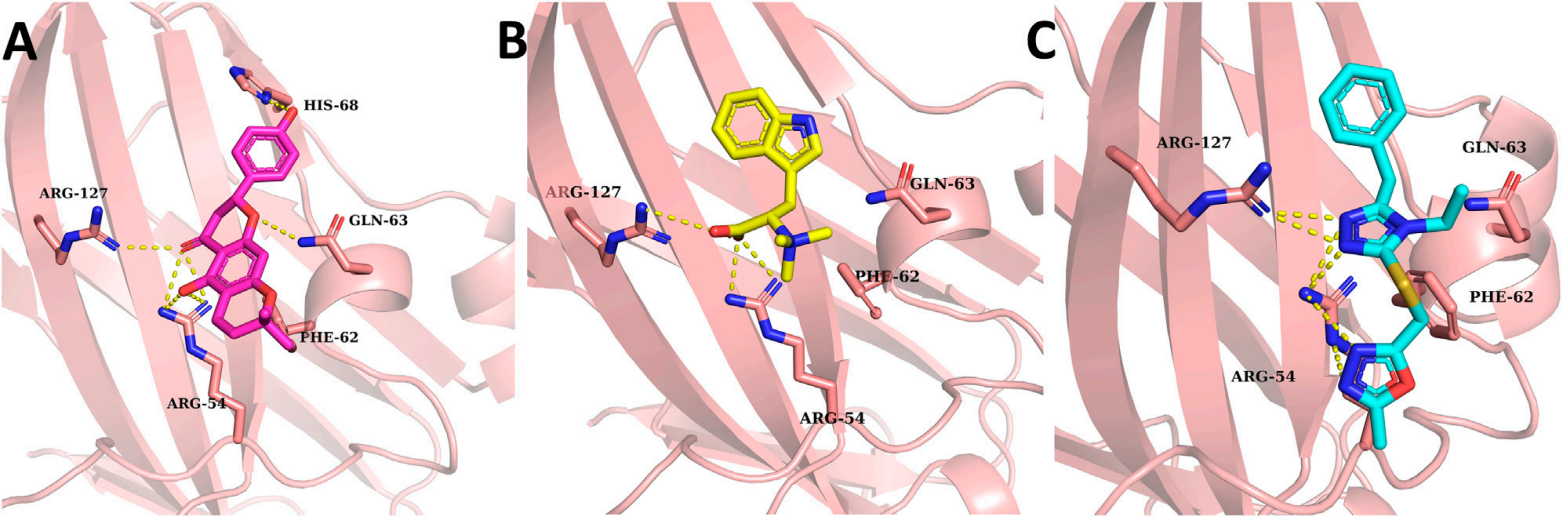
Binding conformation and hydrogen binding residues of the top three compounds at the active site of VISTA (light pink). **(A)** 14187087 (dark pink), **(B)** 3861164 (yellow), and **(C)** 6494266 (cyan). Meanwhile, hydrogen bonding between residues is represented by yellow-colored dotted lines.

The molecular docking approach, one of the reliable approaches in the drug discovery process, was used to determine the natural inhibitors of VISTA protein. Docked ligands were graded based on their binding energy scores. The pose with the lowest score compared to CA-170 was regarded as the stable binding mode of the ligand. The top 20 compounds were visually analyzed using PyMol; out of those 20, three compounds were selected based on the binding conformation and interactions with the active site key residues. These top selected natural ligands were successfully docked in the target active site. The ligand binding poses are depicted in Figure 3.

All the compounds had at least one hydrogen bond with the critical active site residues. Among all three ligands’ compounds, 14187087 has a greater number of hydrogen bonds with an energy value of −6.3 kcal/mol. It formed hydrogen bonds with Arg54, Gln63, His68, and Arg127 residues, out of which two residues (Arg54 and Gln63) are important active site residues. Compounds 3861164 and 6494266 formed two hydrogen bonds with Arg54 and Arg127 with the binding scores −6.8 kcal/mol and −6.7 kcal/mol, respectively (Table 1). All the ligands have two common interactions with Arg54 and Arg127. As the compounds revealed favorable docking scores and good atomic-level chemical interactions, including hydrogen bonding with the VISTA, dynamics supported by binding free estimation were undertaken to further investigate the applicability of these compounds as effective VISTA inhibitors.

**TABLE 1:** Filtered best affinity binders of VISTA. The binding affinity score, total number of hydrogen bonds, and VISTA residues involved in hydrogen bonding with the compound.

PubChem ID	Energy score (kcal/mol)	Total number of hydrogen bonds	Interacting residues with hydrogen bonding
14187087	−6.3	7	Arg54, Gln63, His68, Arg127
3861164	−6.8	3	Arg54, Arg127
6494266	−6.7	6	Arg54, Arg127
CA170	−5.4	9	Tyr37, Gln63, Arg127, Arg54, Tyr41

### Molecular Dynamics Simulation Analysis of the Docked Complexes

All atom MD simulation was conducted using the AMBER package to assess the validity of the docking data and results by analyzing the dynamics behavior of protein atoms and the stability of the compounds at the binding site. For a time scale of 50 ns, the systems were analyzed for structure stability using RMSD and RMSF. The CPPTRAJ module of AMBER 18 was used to calculate the RMSD values to determine the convergence of the trajectories. RMSF values were calculated to determine the structure flexibilities of protein. Even though docking studies have been used effectively for calculating the ligand binding pose for several proteins, they failed to assess the ligand binding affinity (Cheng et al., 2012). During docking, proteins are treated as rigid molecules which do not consider the conformational changes that occur due to the ligand binding (Heitz and Van Mau, 2002). These conformational changes can be studied using molecular dynamics simulations. MD simulations have been extensively used to study the conformational changes in the protein–ligand interactions (Li et al., 2011).

**FIGURE 4:**
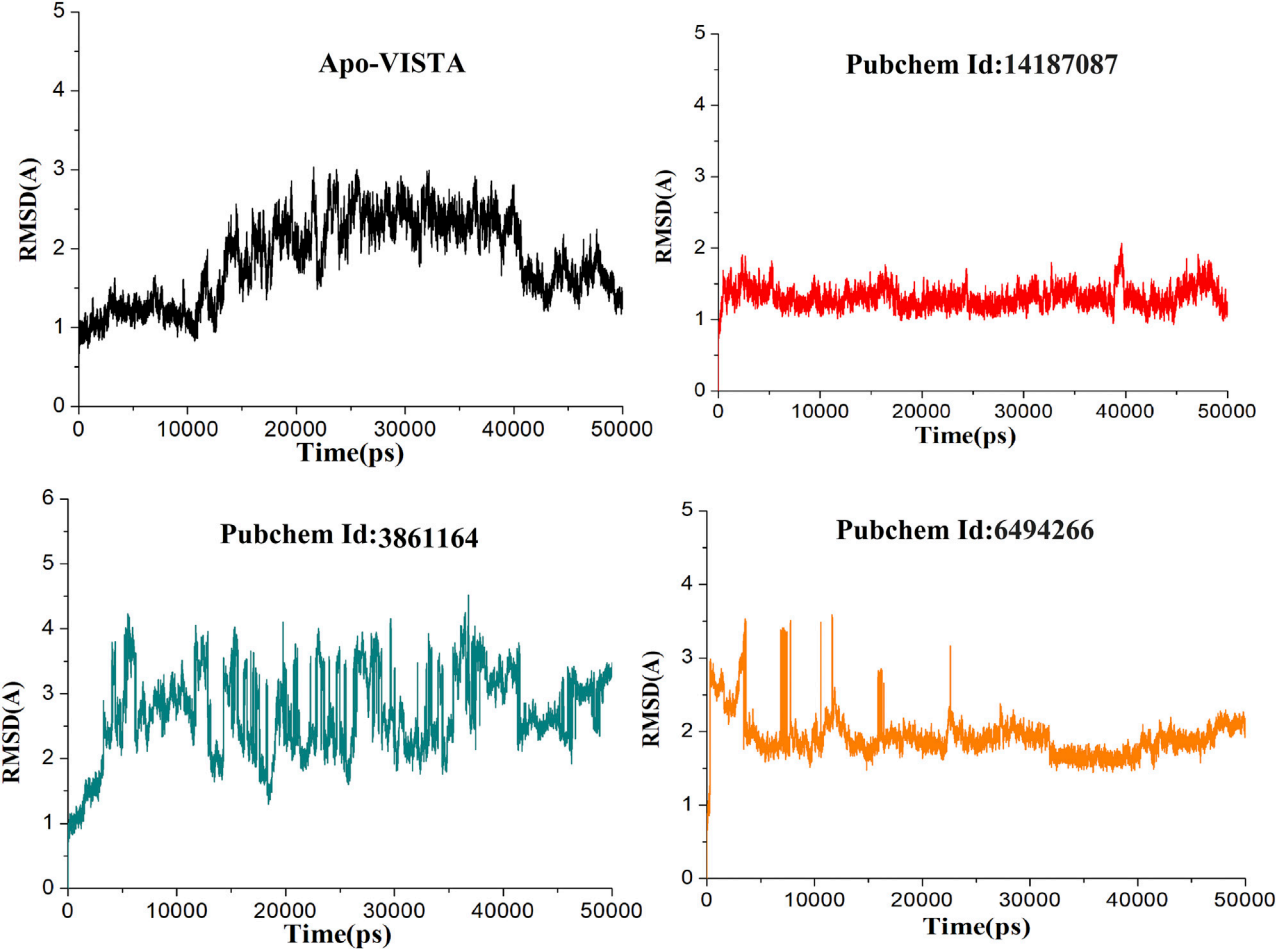
RMSD of all simulated systems as a function of time (Apo-VISAT: black, PubChem ID: 14187087-VISTA: red, PubChem ID: 3861164-VISTA: green, and PubChem ID: 6494266-VISTA: orange).

Compound 6494266 fluctuated up to 3.5 Å during the first 5 ns, but later, after 15 ns, it reached equilibrium. Among all the complexes, the 14187087 compound was the most stable complex throughout the simulation with an average RMSD of 1.31 Å. However, the 3861164 complex kept oscillating throughout the simulation, indicating that this complex might be unstable among all the complexes. Thereafter, the 14187087 and 6494266 complexes were stabilized and showed steady state dynamic behavior, as shown in Figure 4.

**FIGURE 5:**
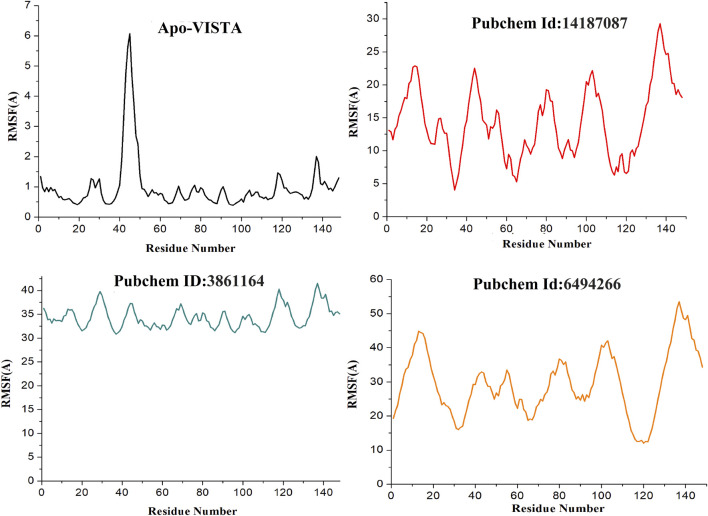
RMSF of simulated systems. The protein residues (on the *x*-axis) are plotted versus their flexibility (on the *y*-axis) from the mean position in simulation time (Apo-VISAT: black, PubChem ID: 14187087-VISTA: red, PubChem ID: 3861164-VISTA: green, and PubChem ID: 6494266-VISTA: orange).

The variability in the conformation of trajectories can be monitored by calculating RMSF for individual atoms. In order to investigate and explore the conformational variability of each trajectory, RMSF of residues was plotted with respect to the residue number to show the local conformational changes for all the simulated complexes (Figure 5). Among all the docked complexes, compound 3861164 and compound 6494266 showed high fluctuations as compared to other systems, which is also consistent with the RMSD results. It can be concluded that the apo-VISTA structure, despite one large peak (40–52 amino acids), is highly stable compared to the VISTA–compound complexes. Conformational rearrangement of the loops than the rest of the protein in the presence of compounds has been previously reported and is linked to greater flexibility (Streaker and Beckett, 1999; Danielson and Lill, 2012). As VISTA protein has higher loop percentage and has a small size, upon ligand binding it is highly likely that loops may behave more dynamically. However, these fluctuations did not disturb the ligand binding conformation and the chemical interaction network, which are key to the stable binding of the compounds throughout the simulation time.

### Analysis of Intermolecular Binding Stability by MM-GBSA

MM-GBSA binding free energies of the complexes were estimated to validate the docking and simulation results. Such MM-GBSA binding free energy is now regularly applied in drug-designing protocols as they are more reliable than conventional docking techniques and less computationally expensive (Alamri et al., 2020a; Alamri et al., 2020b). The binding energies of complexes are presented in Table 2. It was observed that van der Waals energy and electrostatic energy dominated chemical interactions between the compounds and VISTA protein and contributed majorly to the total energy. In the interaction of 14187087, the van der Waals and electrostatic energy values were −32.2723 and −49.3294 kcal/mol, respectively, suggesting that electrostatic interactions were the major forces in the binding of VISTA and compound-1. In the case of 3861164, the contribution of van der Waals energy was −21.4642 kcal/mol and that of electrostatic energy was −16.8891 kcal/mol. In the case of complex 6494266, van der Waals and electrostatic energy was −27.7207 and −37.0189 kcal/mol, respectively. Among all three complexes, 14187087 had the minimum binding energy, indicating it as an effective inhibitor.

**TABLE 2:** Binding free energy calculations of all three complexes. ∆Egas, molecular mechanics energy in the gas phase; ∆Eele, electrostatic energy; ∆Evdw, van der Waals potential energy; ∆Gsol, solvation free energy; ∆Gbind, binding energy.

Energy kcal/mol	14187087	3861164	6494266
∆Evdw	−32.27 ± 4.50	−21.46 ± 6.16	−27.72 ± 3.30
∆Eele	−49.33 ± 14.30	−16.88 ± 9.93	−37.01 ± 11.80
∆Egas	−81.60 ± 16.75	−38.35 ± 14.14	−64.74 ± 11.76
∆Gsol	48.27 ± 9.01	24.68 ± 9.14	43.76 ± 9.08
∆Gbind	−33.33 ± 9.97	−13.67 ± 6.38	−20.97 ± 4.18

### Computational Prediction of Compound Pharmacokinetics

**FIGURE 6:**
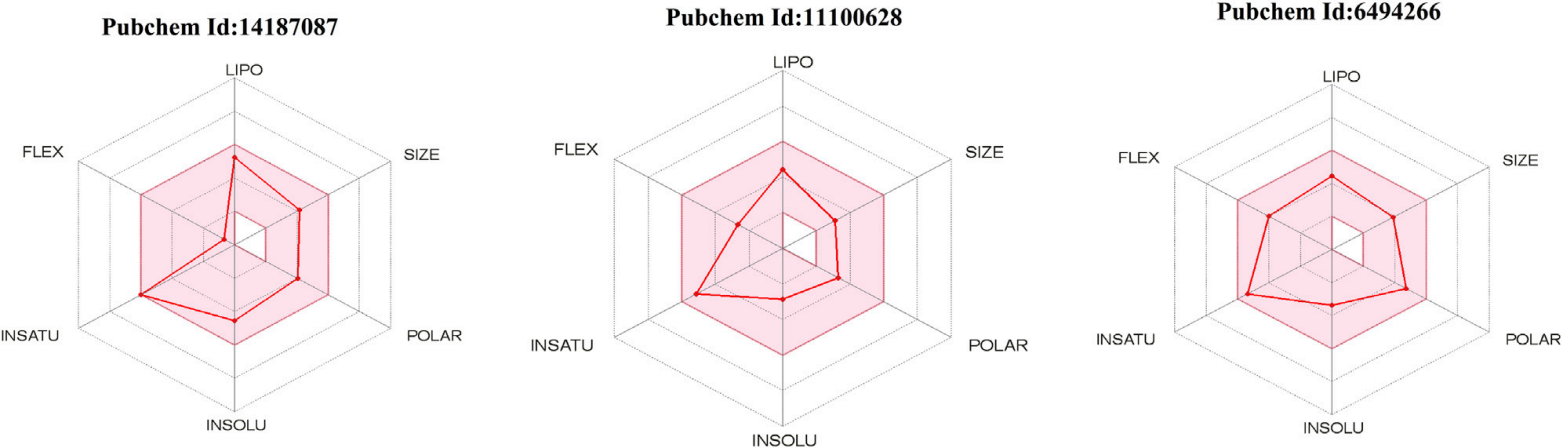
Radar of oral bioavailability (shown by red line). The pink-colored zone presents the physicochemical space allowed for orally bioavailable drugs. INSATU (instauration), INSOLU (insolubility), LIPO (lipophility), POLAR (polarity), FLEX (flexibility), and SIZE (molecular weight).

SwissADME is an online server for calculating different physical and chemical indicators and predicting drug-like properties, ADME parameters, pharmacochemical friendliness, and pharmacokinetic properties to help drug discovery. Detailed results of all the compounds are listed in Table 3. The oral bioavailability radar of the compounds is shown in Figure 6.

**TABLE 3:** Overview of different physicochemical properties, pharmacokinetics, medicinal chemistry, and drug-likeness of the compounds.

Physicochemical properties		Pharmacokinetics	
**PubChem ID: 14187087**			
Formula	C20H18O5	GI absorption	High
Molecular weight	338.35 g/mol	BBB permeant	Yes
Number of heavy atoms	25	P-gp substrate	Yes
Number of arom. heavy atoms	12	CYP1A2 inhibitor	Yes
Fraction Csp3	0.25	CYP2C19 inhibitor	Yes
Number of rotatable bonds	1	CYP2C9 inhibitor	Yes
Number of H-bond acceptors	5	CYP2D6 inhibitor	Yes
Number of H-bond donors	2	CYP3A4 inhibitor	Yes
Molar refractivity	93.67	Log *K*_p_ (skin permeation)	−5.76 cm/s
TPSA	75.99 Å^2^		
Lipophilicity		Drug-likeness	
Log *P*_o/w_ (iLOGP)	2.97	Lipinski	Yes; 0 violation
Log *P*_o/w_ (XLOGP3)	3.67	Ghose	Yes
Log *P*_o/w_ (WLOGP)	3.56	Veber	Yes
Log *P*_o/w_ (MLOGP)	1.82	Egan	Yes
Log *P*_o/w_ (SILICOS-IT)	3.44	Muegge	Yes
Consensus log *P*_o/w_	3.09	Bioavailability score	0.55
Water solubility		Medicinal chemistry	
Log *S* (ESOL)	−4.54	PAINS	0 alert
Solubility	9.78e-03 mg/ml; 2.89e-05 mol/L	Brenk	1 alert: quaternary_nitrogen_2
Class	Moderately soluble	Lead-likeness	No; 1 violation: XLOGP3>3.5
Log *S* (Ali)	−4.96	Synthetic accessibility	3.97
Solubility	3.75e-03 mg/ml; 1.11e-05 mol/L		
Class	Moderately soluble		
Log *S* (SILICOS-IT)	−4.83		
Solubility	5.04e-03 mg/ml; 1.49e-05 mol/L		
Class	Moderately soluble		
**PubChem ID: 3861164**			
Formula		GI absorption	High
	C14H18N2O2		
Molecular weight	246.30 g/mol	BBB permeant	No
Number of heavy atoms	18	P-gp substrate	Yes
Number of arom. heavy atoms	9	CYP1A2 inhibitor	No
Fraction Csp3	0.36	CYP2C19 inhibitor	No
Number of rotatable bonds	4	CYP2C9 inhibitor	No
Number of H-bond acceptors	2	CYP2D6 inhibitor	No
Number of H-bond donors	1	CYP3A4 inhibitor	No
Molar refractivity	69.50	Log *K*_p_ (skin permeation)	−6.23 cm/s
TPSA	55.92 Å^2^		
Lipophilicity		Drug-likeness	
Log *P*_o/w_ (iLOGP)	−1.65	Lipinski	Yes; 0 violation
Log *P*_o/w_ (XLOGP3)	2.21	Ghose	Yes
Log *P*_o/w_ (WLOGP)	0.54	Veber	Yes
Log *P*_o/w_ (MLOGP)	−2.31	Egan	Yes
Log *P*_o/w_ (SILICOS-IT)	1.94	Muegge	Yes
Consensus log *P*_o/w_	0.14	Bioavailability score	0.55
Water solubility		Medicinal chemistry	
Log *S* (ESOL)	−2.87	PAINS	0 alert
Solubility	3.36e-01 mg/ml; 1.36e-03 mol/L	Brenk	1 alert: quaternary_nitrogen_2
Class	Soluble	Lead-likeness	No; 1 violation: MW < 250
Log *S* (Ali)	−3.02	Synthetic accessibility	2.41
Solubility	2.36e-01 mg/ml; 9.58e-04 mol/L		
Class	Soluble		
Log *S* (SILICOS-IT)	−4.33		
Solubility	1.14e-02 mg/ml; 4.65e-05 mol/L		
Class	Moderately soluble		
**PubChem ID:6494266**			
Formula	C15H17N5OS	GI absorption	High
Molecular weight	315.39 g/mol	BBB permeant	No
Number of heavy atoms	22	P-gp substrate	Yes
Number of arom. heavy atoms	16	CYP1A2 inhibitor	Yes
Fraction Csp3	0.33	CYP2C19 inhibitor	Yes
Number of rotatable bonds	6	CYP2C9 inhibitor	Yes
Number of H-bond acceptors	5	CYP2D6 inhibitor	No
Number of H-bond donors	0	CYP3A4 inhibitor	Yes
Molar refractivity	84.57	Log *K*_p_ (skin permeation)	−6.60 cm/s
TPSA	94.93 Å^2^		
Lipophilicity		Drug-likeness	
Log *P*_o/w_ (iLOGP)	2.97	Lipinski	Yes; 0 violation
Log *P*_o/w_ (XLOGP3)	2.29	Ghose	Yes
Log *P*_o/w_ (WLOGP)	2.72	Veber	Yes
Log *P*_o/w_ (MLOGP)	1.86	Egan	Yes
Log *P*_o/w_ (SILICOS-IT)	2.94	Muegge	Yes
Consensus log *P*_o/w_	2.52	Bioavailability score	0.55
Water solubility		Medicinal chemistry	
Log *S* (ESOL)	−3.38	PAINS	0 alert
Solubility	mg/ml; 4.17e-04 mol/l	Brenk	0 alert
Class	Soluble	Lead-likeness	
			Yes
Log *S* (Ali)	−3.92	Synthetic accessibility	3.24
Solubility	3.78e-02 mg/ml; 1.20e-04 mol/l		
Class	Soluble		
Log *S* (SILICOS-IT)	−5.62		
Solubility	7.53e-04 mg/ml; 2.39e-06 mol/l		
Class	Moderately soluble		

The physicochemical properties of the compounds are within the scope of drug-likeness and do not violate any Lipinski rule parameter. In addition, the compounds have good lipophilicity, so they can be transported to the maximum extent and reach the target site (Arnott and Planey, 2012). The compounds were also demonstrated to fulfill all requirements of the prominent Lipinski (Lipinski, 2004), Egan (Egan et al., 2000), Muegge (Muegge et al., 2001), and Veber (Veber et al., 2002) drug-ability rules. The compounds were predicted to be soluble and thus can be good candidates for oral administration. All the compounds were also predicted to not contain pan-assay interference compounds (PAINS) alerts and will not interact nonspecifically with multiple biological targets. This analysis revealed that the screened hits are VISTA-specific and will not have off-target effects. The compounds also have good gastrointestinal absorption, thus indicating that the good concentration of the drugs can reach the target site for performing the required action. Also, the compounds have good synthetic accessibility scores; therefore, they are easy to synthesize for experimental studies.

## Conclusion

The study short-listed Paratocarpin K (PubChem ID: 14187087), 3-(1H-Indol-3-yl)-2-(trimethylazaniumyl)propanoate (PubChem ID: 3861164), and 2-[(5-Benzyl-4-ethyl-1,2,4-triazol-3-yl)sulfanylmethyl]-5-methyl-1,3,4-oxadiazole (PubChem ID: 6494266) from the MPD3 database as effective natural lead inhibitory molecules against VISTA protein, which is an immune checkpoint protein and is considered as a potential therapeutic target, especially for treating triple-negative breast cancer. These molecules unveiled good binding affinity as predicted by the docking technique and showed stable binding modes at the active pocket of VISTA protein. The compounds’ docked conformation dynamics study validated their stable binding nature and compounds remained intact at the active site by both hydrophobic and hydrophilic interactions with key active residues of the protein. Additionally, confirmation on the binding stability of the compounds was accomplished through the binding free energy approach, which also revealed consistent results with the docking and MD simulation outcomes. The study employed a comprehensive computational framework to identify anticancer molecules by targeting VISTA protein. Although each step is thoroughly validated and the results are investigated for accuracy *via* follow-up computational approaches, the study suffers from lack of experimental validation. Altogether, the study findings are promising and could be subjected to further experimental evaluation to disclose their anti-VISTA/cancer potency.

## Data Availability

The original contributions presented in the study are included in the article/Supplementary Material; further inquiries can be directed to the corresponding authors.
